# Insights into the recent emergence and expansion of eastern equine encephalitis virus in a new focus in the Northern New England USA

**DOI:** 10.1186/s13071-015-1145-2

**Published:** 2015-10-09

**Authors:** Goudarz Molaei, Philip M. Armstrong, Alan C. Graham, Laura D. Kramer, Theodore G. Andreadis

**Affiliations:** Center for Vector Biology & Zoonotic Diseases, The Connecticut Agricultural Experiment Station, 123 Huntington Street, New Haven, CT 06511 USA; Vermont Agency of Agriculture, 322 Industrial Lane, Barre, VT 05641 USA; Wadsworth Center, New York State Department of Health, 5668 State Farm Rd, Slingerlands, NY 12159 USA

**Keywords:** *Culiseta melanura*, Eastern equine encephalitis virus, Transmission, Blood-feeding, Vermont

## Abstract

**Background:**

Eastern equine encephalomyelitis virus (EEEV) causes a highly pathogenic zoonosis that circulates in an enzootic cycle involving the ornithophagic mosquito, *Culiseta melanura*, and wild passerine birds in freshwater hardwood swamps in the northeastern U.S. Epidemic/epizootic transmission to humans/equines typically occurs towards the end of the transmission season and is generally assumed to be mediated by locally abundant and contiguous mammalophagic “bridge vector” mosquitoes.

**Methods:**

Engorged mosquitoes were collected using CDC light, resting box, and gravid traps during epidemic transmission of EEEV in 2012 in Addison and Rutland counties, Vermont. Mosquitoes were identified to species and blood meal analysis performed by sequencing mitochondrial *cytochrome b* gene polymerase chain reaction products. Infection status with EEEV in mosquitoes was determined using cell culture and RT-PCR assays, and all viral isolates were sequenced and compared to other EEEV strains by phylogenetic analysis.

**Results:**

The host choices of 574 engorged mosquitoes were as follows: *Cs. melanura* (*n* = 331, 94.3 % avian-derived, 5.7 % mammalian-derived); *Anopheles quadrimaculatus* (*n* = 164, 3.0 % avian, 97.0 % mammalian); *An. punctipennis* (*n* = 56, 7.2 % avian, 92.8 % mammalian), *Aedes vexans* (*n* = 9, 22.2 % avian, 77.8 % mammalian); *Culex pipiens* s.l. *n* = 6, 100 % avian); *Coquillettidia perturbans* (*n* = 4, 25.0 % avian, 75.0 % mammalian); and *Cs. morsitans* (*n* = 4, 100 % avian). A seasonal shift in blood feeding by *Cs. melanura* from Green Heron towards other avian species was observed. EEEV was successfully isolated from blood-fed *Cs. melanura* and analyzed by phylogenetic analysis. Vermont strains from 2012 clustered with viral strains previously isolated in Virginia yet were genetically distinct from an earlier EEEV isolate from Vermont during 2011.

**Conclusions:**

*Culiseta melanura* acquired blood meals primarily from birds and focused feeding activity on several competent species capable of supporting EEEV transmission. *Culiseta melanura* also occasionally obtained blood meals from mammalian hosts including humans. This mosquito species serves as the primary vector of EEEV among wild bird species, but also is capable of occasionally contributing to epidemic/epizootic transmission of EEEV to humans/equines. Other mosquito species including *Cq. perturbans* that feed more opportunistically on both avian and mammalian hosts may be important in epidemic/epizootic transmission under certain conditions. Phylogenetic analyses suggest that EEEV was independently introduced into Vermont on at least two separate occasions.

## Background

Eastern Equine Encephalitis virus (EEEV) (*Alphavirus: Togaviridae*) is a highly pathogenic virus that circulates in an enzootic cycle involving the ornithophagic mosquito, *Culiseta melanura* (Coquillett) and wild passerine (perching) birds in mostly freshwater hardwood swamps in the northeastern United States [1–6]. Historically, epizootics in equines and epidemics in humans occurred intermittently; however, during the last decade, we have observed increases in the frequency and amplitude of virus activity, and a northward expansion of its geographic range [7]. Increased virus detection has been reported from the northeastern states: New York, New Hampshire, and Massachusetts, and more recently from Vermont, Connecticut, and Maine [7–18].

Vermont is a newly emergent region for EEEV activity that is located on the northern boundary of the geographic range of the virus. EEEV was first isolated in Vermont during a 2011 outbreak on an Emu, *Dromaius novaehollandiae* (Latham), farm in Rutland County [18]. The first confirmed human cases of EEE occurred in the state during the subsequent year and the virus was also detected in *Cs. melanura* pools from the same region of Vermont [18]. Neutralizing antibodies to EEEV were also found during a serosurvey of white-tailed deer, *Odocoileus virginianus* (Zimmermann), and moose, *Alces americanus* (Clinton), sampled throughout Vermont [19, 20]. Otherwise, little is known concerning the ecology of the virus in this region of the U.S.

*Culiseta melanura* is widely recognized as a principal vector of EEEV in enzootic cycling among wild birds; however, its potential contribution to epidemic/epizootic transmission to humans/equines is not clearly understood. Recent studies indicate that a small percentage of these mosquitoes will occasionally feed on mammalian hosts, thus qualifying this species as a potential vector to humans/equines [17, 21, 22]. Other mosquito species, such as *Aedes vexans* (Meigen), *Coquillettidia perturbans* (Walker)*, Aedes canadensis* (Theobald), and *Aedes sollicitans* (Walker) with tendencies to primarily feed on mammals, or opportunistically on both avian and mammalian hosts, have also been implicated with epidemic/epizootic transmission of EEEV on the basis of their vector competence, local abundance, geographic distribution, host feeding patterns, and/or virus isolation during epidemics [8, 14, 21–29].

The current research initiative was undertaken to 1) characterize the host-feeding patterns of *Cs. melanura* and evaluate its role in enzootic maintenance of EEEV in wild bird populations during an epidemic in Vermont, 2) assess the potential involvement of *Cs. melanura* in epidemic/epizootic transmission to humans and equines, and 3) identify key avian species as hosts for mosquitoes which also support amplification of EEEV. Accordingly, blood-fed mosquitoes were collected from EEEV transmission foci in Addison and Rutland counties, Vermont, and analyzed for the host source by sequencing mitochondrial *cytochrome b* gene. We report the isolation of EEEV from *Cs. melanura* with the identification of the vertebrate host species on which they had fed. Phylogenetic analysis was also conducted to compare and evaluate the relationships among EEEV isolates from mosquitoes in the region and gain insights into the temporal and spatial introduction of EEEV to Vermont.

## Methods

### Study area

**Fig. 1:**
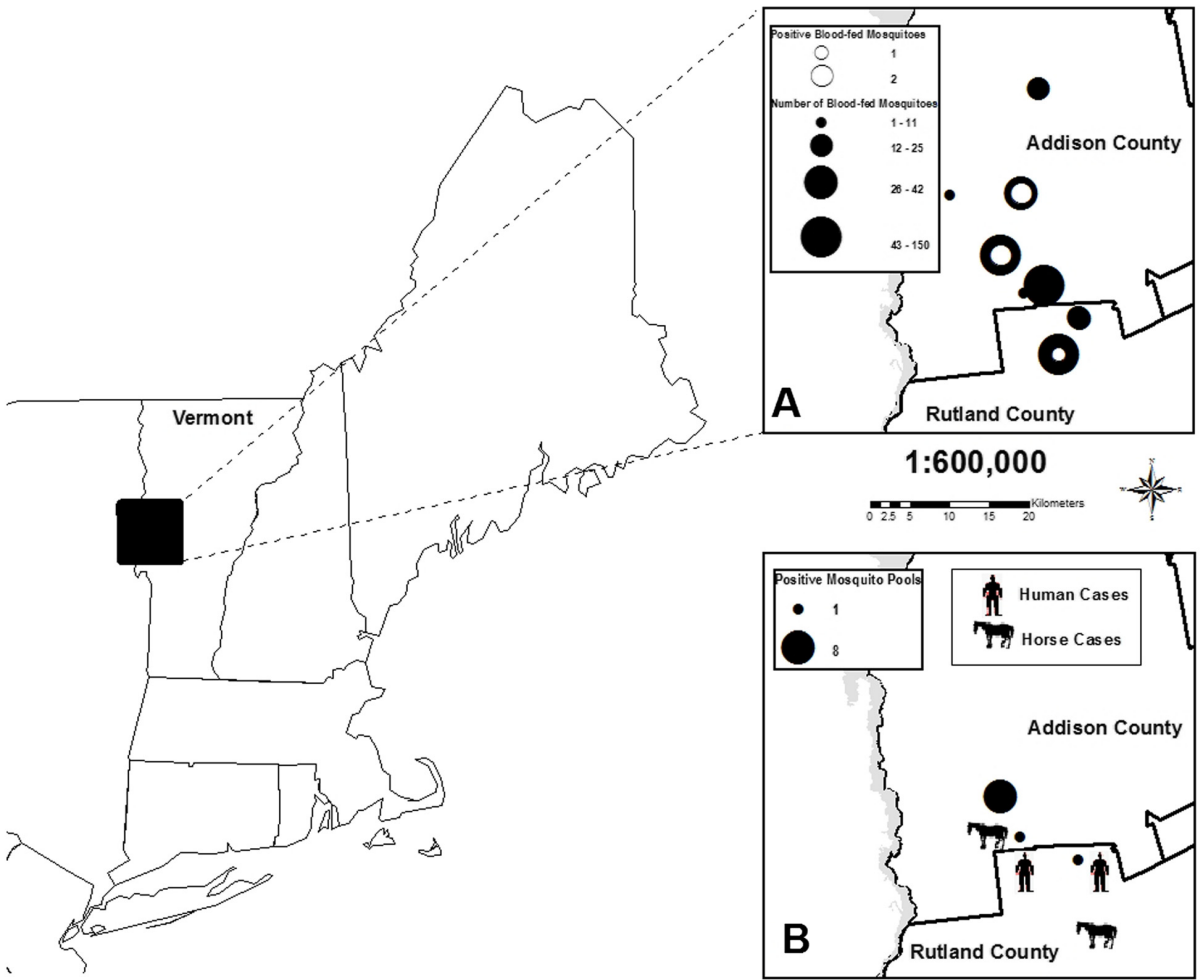
Data collected in Addison and Rutland Counties, Vermont, June through October, 2012. **a** Close-up of Addison and Rutland Counties, VT with number of EEEV positive blood-fed *Cs. melanura* in open circles and number of blood-fed mosquitoes in closed circles. **b** Close-up of Addison and Rutland Counties, VT with number of positive mosquito pools, and human and equine infections

Study was conducted in Addison and Rutland counties, Vermont (Fig. 1). Addison County has a total area of 2,090 km^2^ with 292 km^2^ (14 %) of wetlands. The county is situated on the west line of Vermont state and nearly in the center north and south; between 43° 50′ and 44° 10′ N. Rutland County has a total area of 2,450 km^2^ with 270 km^2^ (11 %) of wetlands. Otter Creek is the primary stream, which runs through the two counties from the south to the north. There are extensive wetlands surrounding the north-flowing Otter Creek basin, subject to periodic flooding. The most dominant hardwood swamp in this region consists of red maple, *Acer rubrum* Linnaeus, and black ash, *Fraxinus nigra* Marshall. Some red maple/ sphagnum swamps have formed in isolated wetlands not influenced by flood events [30]. These acidic hardwood swamps are also suitable habitats not only for plants preferring acidic conditions, but also for *Cs. melanura*, the primary vector of EEEV.

### Mosquito collection

Mosquitoes were collected June 16 through October 22, 2012. CDC light and black plastic Coroplast resting box traps (Mills Industries, Inc. Laconia, NH) were used for collecting mosquitoes. Gravid traps were also placed in the town of Brandon, which has a history of virus activity, where EEEV was isolated from a flock of emus in 2011 [18].

### Mosquito specimen processing and morphological identification

Identification of mosquitoes to species was carried out by using a morphological key with the aid of a dissecting microscope on chill tables or dry ice [31]. All mosquitoes with visible sign of blood were placed in 2.0 ml tubes and stored in an ultra-low temperature freezer for subsequent blood meal analysis and virus testing.

### DNA isolation and blood meal analyses

Mosquito abdomens were removed for blood meal analysis using disposable razor blades. DNA was extracted from the abdomen of blood-fed mosquitoes using DNAzol BD (Molecular Research Center, Cincinnati, OH) according to the manufacturer’s recommendation with modifications as described elsewhere [21, 32, 33]. Isolated DNA from the mosquito blood meals was used as a DNA template in subsequent polymerase chain reaction (PCR) assays with primers based on mitochondrial *cytochrome b* sequences of avian and mammalian species. The thermal cycling conditions were as described earlier [21, 32, 33]. The Veriti Dx Thermal Cycler, or GeneAmp PCR System 9700 (Applied Biosystems, Foster City, CA) were used to perform PCR assays, and PCR products were sequenced directly in cycle sequencing reactions using the sequencer 3730xl DNA Analyzer (Applied Biosystems) at the Keck Sequencing Facility (Yale University, New Haven, CT). Sequence annotation and analyses were conducted using ChromasPro version 1.7.5 (Technelysium Pty Ltd., Tewantin, Australia), and compared to the sequences available at the GenBank DNA sequence database of the National Center for Biotechnology Information using the BLAST search (BLASTN) [34].

### Virus testing of mosquitoes

The head and thorax of blood-fed mosquitoes were individually homogenized in 0.5 mL phosphate buffered saline (PBS) containing 30 % heat-inactivated rabbit serum, 0.5 % gelatin, and 1X antibiotic/antimycotic using a copper BB and vibration mill. Mosquito homogenates were centrifuged at 4 °C for 7 min at 7,000 rpm and 100 μL of the supernatant were inoculated onto a monolayer of confluent Vero cells. Cells were maintained at 37 °C in 5 % CO_2_ and examined daily for cytopathic effect (CPE) from day 3 through day 7 following inoculation. RNA was extracted from CPE-positive cultures using the QIAamp viral RNA kit and tested for EEEV by TaqMan assay as previously described [35].

Non-blooded mosquitoes were grouped into pools of 50 or fewer individuals and tested by real-time RT-PCR and inoculation of Vero cells [36, 37]. Briefly, mosquitoes were homogenized in tissue culture diluent, centrifuged at 4 °C for 3 min at 13,000 rpm. One aliquot was removed for virus isolation on Vero cell culture, and another for storage. Lysis buffer was added to the remainder for RNA extraction, followed by purification from the extract by Tecan EVO Freedom automated system (Tecan Group Ltd., Männedorf, Switzerland), and MagMax 96 Viral Isolation Kit (Applied Biosystems).

### Virus sequencing and phylogenetic analysis

EEEV sequence was derived from four overlapping PCR products spanning the entire coding region and flanking portions of the 5′ and 3′ untranslated regions of the virus genome. RT-PCR was performed using the Titan One-Tube RT-PCR System (Roche Diagnostics, Indianapolis, IN) and one of the following primer pairs:

EEE1 FWD 5′-ATA GGG TAC GGT GTA GAG GCA AC-3′ and

EEE3191 REV 5′-AGG CCA TCT CAG GCG AAT AC-3′;

EEE3085 FWD 5′-AGC GCT AGA ACC TGT GTT GG-3′ and

EEE6258 REV 5′-GAT CGT ATC TCA GGC CGC AA-3′;

EEE6162 FWD 5′-ACG GAG CAT CCT GCT GTT TA-3′ and

EEE9119 REV 5′-CCT TGG CAC TGT GGA TGC TA-3′;

EEE8859 FWD 5′-ACA TCT TGG CTC AAT GCC CA-3′ and

EEE11430 REV 5′-ATG CAC CAC CGT CAC CAT AG-3′

PCR products were amplified under the following thermal cycling conditions: one cycle of 48 °C for 30 min and 94 °C for 2 min, 10 cycles of 94 °C for 15 s, 58 °C for 30 s, and 68 °C for 2 min 30 s, followed by 25 cycles of 94 °C for 15 s, 58 °C for 30 s, and 68 °C for 2 min 30 s + 5 s per cycle, and 1 cycle of 68 °C for 7 min. PCR products were purified using the PCR purification kit (Qiagen , Valencia, CA) and sequenced (Yale DNA Analysis Facility, New Haven, CT) using 27 sequencing primers (sequences available upon request).

Overlapping sequence chromatograms were edited using ChromasPro version 1.7.5 (Technelysium Pty Ltd.) and edited sequences were deposited in GenBank (Accession no. KT153580 and KT153581). Multiple sequence alignments were generated using the ClustalW algorithm, and the resulting alignment was comprised of 58 viruses and 11,104 nucleotide sites [38]. Phylogenetic and molecular evolutionary analyses were conducted by maximum-likelihood (ML) analysis using MEGA version 6 [39]. The optimal nucleotide substitution model was selected by performing ML fits of 24 different models in MEGA. Support for individual nodes was evaluated by performing 1000 bootstrap replicates.

### Avian population abundance estimates

**Fig. 2:**
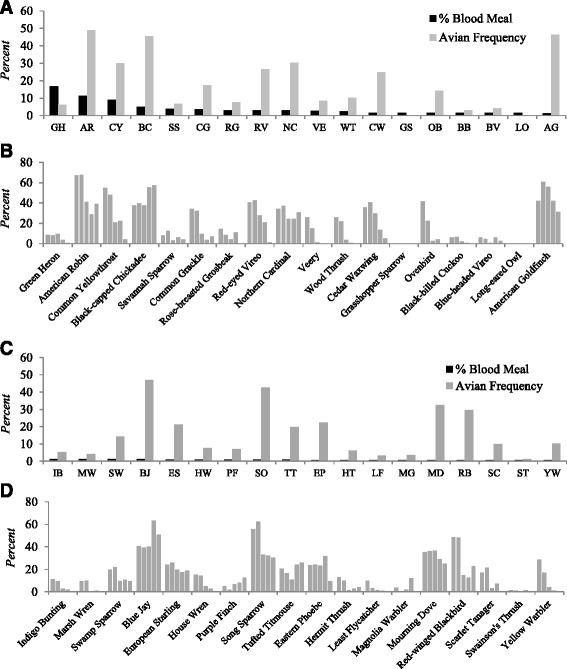
**a** and **c** Percentage of avian-derived blood meals for *Cs. melanura* compared with average avian frequencies in Addison and Rutland Counties, VT, June through October, 2012. **b** and **d** Monthly frequencies of avian species based on point count data in Addison and Rutland Counties, VT, June through October, 2012. Bars above each avian species represent frequencies of these species from June through October, during which blood-fed mosquitoes had also been collected

Observation frequency of local avian species (Fig. 2) were estimated for 5 months from June through October 2012, based on the information available through a project by the Cornell Laboratory of Ornithology and the National Audubon Society to track the bird abundance in North America. “Frequency” represents the percentage of checklists reporting the species within a specified date range and region. Observation frequency of avian populations was expressed in decimal format ranging from 0 to 1, indicating “absent” to “present” for all observations. The frequency data consist of information obtained from historic submissions of bird count lists from birders in and around the wetlands where blood-fed mosquitoes were collected [40].

## Results

### Mosquito species

Vertebrate hosts of 574 engorged field-collected mosquitoes of seven species, *Cs. melanura* (*n* = 331), *An. quadrimaculatus* Say (*n* = 164), *An. punctipennis* (Say) (*n* = 56), *Ae. vexans* (*n* = 9); *Cx. pipiens* s.l. (*n* = 6), *Coquillettidia perturbans*, and *Culiseta morsitans* (Theobald) (each *n* = 4) were identified to species.

### Vertebrate host choice by mosquitoes

#### *Culiseta melanura*

Of the 331 engorged *Cs. melanura*, 312 (94.3 %) had avian- and 19 (5.7 %) mammalian-derived blood meals (Table 1). Forty-nine avian species representing 18 families and 5 orders were identified (Tables 1 and 2). Passeriformes constituted the most numerous hosts representing 77.6 % of avian-derived blood meals. Ciconiiformes represented the second most frequent source (18.3 %), whereas comparatively few Cuculiformes (1.9 %), Strigiformes (1.6 %), and Columbiformes (0.6 %) were identified (Table 2). Green Heron, *Butorides virescens* (L.) was the most frequent host (16.9 % of all vertebrate hosts), followed by American Robin, *Turdus migratorius* L. (11.5 %), Common Yellowthroat, *Geothlypis trichas* (L.) (9.1 %), Black-capped Chickadee, *Poecile atricapillus* (L.) (5.1 %), and then 45 other avian species (51 %) (Table 1).

**Table 1:** Number and percentage of avian- and mammalian-derived blood meals identified from *Culiseta melanura* in Vermont, June through October, 2012

Vertebrate Host	Scientific Name	R. C.	June	July	Aug	Sept	Oct	Total (%)
Avian								
Green Heron	*Butorides virescens*	S	28	28				56 (16.9)
American Robin	*Turdus migratorius*	S	5	8	19	6		38 (11.5)
Common Yellowthroat	*Geothlypis trichas*	S	1	4	7	16	2	30 (9.1)
Black-capped Chickadee	*Poecile atricapillus*	P	2	7	5	2	1	17 (5.1)
Savannah Sparrow	*Passerculus sandwichensis*	S		2	3	7	1	13 (3.9)
Common Grackle	*Quiscalus quiscula*	S	1	4	2	5		12 (3.6)
Rose-breasted Grosbeak	*Pheucticus ludovicianus*	S		1	1	8		10 (3.0)
Red-eyed Vireo	*Vireo olivaceus*	S		3	2	5		10 (3.0)
Northern Cardinal	*Cardinalis cardinalis*	P		4	5	1		10 (3.0)
Veery	*Catharus fuscescens*	S	1	4	3	1		9 (2.7)
Wood Thrush	*Hylocichla mustelina*	S		3	3	2		8 (2.4)
Cedar Waxwing	*Bombycilla cedrorum*	P		2	2	2		6 (1.8)
Grasshopper Sparrow	*Ammodramus savannarum*	S	1	1	4			6 (1.8)
Ovenbird	*Seiurus aurocapilla*	S		3	2	1		6 (1.8)
Black-billed Cuckoo	*Coccyzus erythropthalmus*	S		1	4			5 (1.5)
Blue-headed Vireo	*Vireo solitarius*	S		4	1			5 (1.5)
Long-eared Owl	*Asio otus*	S			3	2		5 (1.5)
American Goldfinch	*Spinus tristis*	P			2	1	1	4 (1.2)
Indigo Bunting	*Passerina cyanea*	S			2	2		4 (1.2)
Marsh Wren	*Cistothrous palustris*	S	1		1	2		4 (1.2)
Swamp Sparrow	*Melospiza georgiana*	S		2	1	1		4 (1.2)
Blue Jay	*Cyanocitta cristata*	P	1	3				4 (1.2)
European Starling	*Sturnus vulgaris*	P		1	1	1		3 (0.9)
House Wren	*Troglodytes aedon*	S		1	2			3 (0.9)
Purple Finch	*Carpodacus purpureus*	P			1	2		3 (0.9)
Song Sparrow	*Melospiza melodia*	S		2	1			3 (0.9)
Tufted Titmouse	*Baeolophus bicolor*	P			3			3 (0.9)
Eastern Phoebe	*Sayornis phoebe*	S				2		2 (0.6)
Hermit Thrush	*Catharus guttatus*	S		1	1			2 (0.6)
Least Flycatcher	*Empidonax minimus*	S		1		1		2 (0.6)
Magnolia Warbler	*Dendroica magnolia*	S			2			2 (0.6)
Mourning Dove	*Zenaida macroura*	S	1	1				2 (0.6)
Red-winged Blackbird	*Agelaius phoeniceus*	S		2				2 (0.6)
Scarlet Tanager	*Piranga olivacea*	S	1		1			2 (0.6)
Swainson’s Thrush	*Catharus ustulatus*	S				2		2 (0.6)
Yellow Warbler	*Dendroica petechia*	S		2				2 (0.6)
American Bittern	*Botaurus lentiginosus*	S		1				1 (0.3)
American Crow	*Corvus brachyrhynchos*	P				1		1 (0.3)
Baltimore Oriole	*Icterus galbula*	S		1				1 (0.3)
Bay-breasted Warbler	*Dendroica castanea*	M				1		1 (0.3)
Black-and-White Warbler	*Mniotilta varia*	S		1				1 (0.3)
Bobolink	*Dolichonyx oryzivorus*	S		1				1 (0.3)
Canada Warbler	*Wilsonia canadensis*	S		1				1 (0.3)
Gray Catbird	*Dumetella carolinensis*	S				1		1 (0.3)
Rusty Blackbird	*Euphagus carolinus*	S	1					1 (0.3)
White-throated Sparrow	*Zonotrichia albicollis*	S					1	1 (0.3)
Willow Flycatcher	*Empidonax traillii*	S	1					1 (0.3)
Yellow-billed Cuckoo	*Coccyzus americanus*	S			1			1 (0.3)
Yellow-rumped Warbler	*Setophaga coronata*	S				1		1 (0.3)
Mammalian								
Cow	*Bos taurus*	P		14				14 (4.2)
Human	*Homo sapiens*	P		4				4 (1.2)
White-tailed Deer	*Odocoileus virginianus*	P		1				1 (0.3)
Total			45	119	85	76	6	331

R. C. = Residency codes: P, permanent resident (found year round in the state); S, summer resident (present in state during the nesting season); M, migratory (migrates through the state in spring and/or fall)

**Table 2:** Number and percentage of avian families (*n* = 18) as host choice for *Culiseta melanura* in Addison and Rutland Counties, Vermont, June through October, 2012

Order/Family	June	July	Aug	Sept	Oct	Total
	No. (%)	No. (%)	No. (%)	No. (%)	No. (%)	No. (%)
*Passeriformes*						
Turdidae (Thrushes)	6 (10.2)	16 (27.1)	26 (44.1)	11 (18.6)		59 (18.9)
Parulidae (Wood Warblers)	1 (2.3)	11 (25.0)	11 (25.0)	19 (43.2)	2 (4.6)	44 (14.4)
Emberizidae (Emberizids)	1 (3.7)	7 (25.9)	9 (33.3)	8 (29.6)	2 (7.4)	27 (8.7)
Cardinalidae (Cardinals and Tanagers)	1 (3.9)	5 (19.2)	9 (34.6)	11 (42.3)		26 (8.3)
Paridae (Chickadees and Titmice)	2 (10.0)	7 (35.0)	8 (40.0)	2 (10.0)	1 (5.0)	20 (6.4)
Icteridae (Blackbirds)	2 (11.8)	8 (47.1)	2 (11.8)	5 (29.4)		17 (5.4)
Vireonidae (Vireos)		7 (46.7)	3 (20.0)	5 (33.3)		15 (4.8)
Fringillidae (Sparrows and Finches)			3 (42.9)	3 (42.9)	1 (14.3)	7 (2.2)
Troglodytidae (Wrens)	1 (14.3)	1 (14.3)	3 (42.9)	2 (28.6)		7 (2.2)
Bombycillidae (Waxwings)		2 (33.3)	2 (33.3)	2 (33.3)		6 (1.9)
Corvidae (Jays and Crows)	1 (20.0)	3 (60.0)		1 (20.0)		5 (1.6)
Tyrannidae (Tyrant Flycatchers)	1 (20.0)	1 (20.0)		3 (60.0)		5 (1.6)
Sturnidae (Starlings)		1 (33.3)	1 (33.3)	1 (33.3)		3 (1.0)
Mimidae (Mockingbirds and Thrashers)				1 (100)		1 (0.3)
*Ciconiiformes*						
Ardeidae (Herons, Bitterns, and Allies)	28 (49.1)	29 (50.9)				57 (18.3)
*Cuculiformes*						
Cuculidae (Cuckoos)		1 (16.7)	5 (83.3)			6 (1.9)
*Strigiformes*						
Strigidae (Typical Owls)			3 (60.0)	2 (40.0)		5 (1.6)
*Columbiformes*						
Columbidae (Doves and Pigeons)	1 (50.0)	1 (50.0)				2 (0.6)
Total	45	100	85	76	6	312

Of the 49 avian species as hosts for *Cs. melanura,* Passeriformes comprised 44 species (89.8 %), followed by Ciconiiformes 2 species (4.1 %), Cuculiformes, Columbiformes and Strigiformes, each one species (2.0 %) (Tables 1 and 2). Mammalian hosts included the domestic cow, *Bos taurus* L. (*n* = 14, 73.7 % of mammalian hosts), human, *Homo sapiens *L. (*n* = 4, 21.1 %), and white-tailed deer (*n* = 1, 5.3 %) (Table 1).

#### *Anopheles quadrimaculatus*

Of the 164 *An. quadrimaculatus*, 159 (97.0 %) obtained blood meals from mammalian species, and 3 (3.0 %) had avian-derived blood meals. Domestic cows were the most frequent host (72.6 % of all vertebrate-derived blood meals) followed by 7 other mammalian species including horse, *Equus caballus* L. (2.4 %), and humans (1.2 %). Avian hosts included the American Robin (0.6 %), Common Yellowthroat (0.6 %), and Emu (1.8 %) (Table 3). *Anopheles quadrimaculatus* mosquitoes that fed on emus were collected from a site in close proximity to an Emu farm.

**Table 3:** Number and percentage of avian- and mammalian-derived blood meals identified from *Ae. vexans, An. punctipennis, An. quadrimaculatus, Cq. perturbans, Cs. morsitans, and Cx. pipiens* s.l. in Vermont, June through October, 2012

Vertebrate Host	Scientific Name	R. C.	*Aedes vexans* No. (%)	*Anopheles punctipennis* No. (%)	*Anopheles quadrimaculatus* No. (%)	*Coquillettidia perturbans* No. (%)	*Culiseta morsitans* No.(%)	*Culex pipiens* s.l. No. (%)	Total
Avian									
Green Heron	*Butorides virescens*	S	1 (11.1)	1 (1.8)				1 (16.7)	3
American Robin	*Turdus migratorius*	S			1 (0.6)				1
Common Yellowthroat	*Geothlypis trichas*	S		1 (1.8)	1 (0.6)				2
Red-eyed Vireo	*Vireo olivaceus*	S		1 (1.8)					1
Northern Cardinal	*Cardinalis cardinalis*	P		1 (1.8)					1
Wood Thrush	*Hylocichla mustelina*	S					4 (100.0)	3 (50.0)	7
Cedar Waxwing	*Bombycilla cedrorum*	P						1 (16.7)	1
Swamp Sparrow	*Melospiza georgiana*	S	1 (11.1)						1
Bobolink	*Dolichonyx oryzivorus*	S						1 (16.7)	1
Emu	*Dromaius novaehollandiae*	P			3 (1.8)				3
Mallard	*Anas platyrhynchos*	P				1 (25.0)			1
Mammalian									
Cow	*Bos taurus*	P	3 (33.4)	10 (17.9)	119 (72.6)				132
Human	*Homo sapiens*	P		3 (5.4)	2 (1.2)				5
White-tailed Deer	*Odocoileus virginianus*	P	4 (44.4)	38 (67.9)	29 (17.7)	3 (75.0)			74
Horse	*Equus caballus*	P		1 (1.8)	4 (2.4)				5
Donkey	*Equus asinus*	P			2 (1.2)				2
Sheep	*Ovis aries*	P			1 (0.6)				1
Goat	*Capra hircus*	P			1 (0.6)				1
Eastern Gray Squirrel	*Sciurus carolinensis*	P			1 (0.6)				1
Total			9	56	164	4	4	6	243

R. C. = Residency codes: P, permanent resident (found year round in the state); S, summer resident (present in state during the nesting season)

#### *Anopheles punctipennis*

Of the 56 blood-fed *An. punctipennis*, 52 (92.8 %) obtained blood meals from mammalian hosts, and 4 (7.2 %) had avian-derived blood meals. White-tailed deer was the most frequent source (67.9 %) followed by domestic cows (17.9 %), humans (5.4 %)*,* and horses (1.8 %). Avian hosts included the Green Heron, Common Yellowthroat, Red-eyed Vireo, *Vireo olivaceous* (L.), and Northern Cardinal, *Cardinalis cardinalis* (L.) (each 1.8 %) (Table 3).

#### *Aedes vexans*

Of the 9 blood-fed *Ae. vexans*, 7 (77.8 %) obtained blood meals from mammalian hosts, and 2 (22.2 %) had avian-derived blood meals. Mammalian hosts of *Ae. vexans* included white-tailed deer (*n* = 4, 44.4 %), and domestic cows (*n* = 3, 33.4 %). Avian hosts were identified as Green Heron, and Swamp Sparrow, *Melospiza georgiana* (Latham) (each *n* = 1, 11.1 %) (Table 3).

#### *Culex pipiens*

All specimens had acquired blood meals from avian hosts including the Wood Thrush, *Hylocichla mustelina* (Gmelin) (*n* = 3, 50 %)), Green Heron, Cedar Waxwing, *Bombycilla cedrorum* Vieillot and the Bobolink, *Dolichonyx oryzivorus* L. (each *n* = 1, 16.7 %) (Table 3).

#### *Culiseta morsitans*

All 4 specimens had obtained blood meals from the avian host, Wood Thrush (Table 3).

#### *Coquillettidia perturbans*

Of the 4 *Cq. perturbans*, 3 (75 %) had mammalian- and 1 (25 %) avian-derived blood meals. All three mammalian blood meals were from white-tailed deer, and the avian host was identified as the Mallard Duck, *Anas platyrhynchos* L. (Table 3).

### Seasonal variation in avian host composition

The monthly prevalence of *Cs. melanura* blood meals acquired from 49 avian species is shown in Table 1. In June, we identified 45 avian-derived blood meals from 13 species, of which 62.2 % (*n* = 28) were from Green Heron, followed by American Robin (*n* = 5, 11.1 %), Black-capped Chickadee (*n* = 2, 4.4 %), and 10 other bird species (*n* = 10, 22.3 %). In July, although the number of avian hosts increased to 31 species, Green Heron remained the most frequently identified host (*n* = 28, 23.5 %), followed by American Robin (*n* = 8, 6.7 %), and then several other species (each *n* = 4, 3.4 %). In August however, no Green Heron-derived blood meals were identified and American Robin was the most frequently identified host (*n* = 19, 22.4 %), followed by Common Yellowthroat (*n* = 7, 8.2 %), Black-capped Chickadee and Northern Cardinal (each *n* = 5, 5.9 %). In September, Common Yellowthroat served as the most frequently identified host (*n* =16, 21.1 %), followed by Rose-breasted Grosbeak, *Pheucticus ludovicianus* L. (*n* = 8, 10.5 %), and several other bird species (*n* = 29, 38.2 %). Only 6 avian-derived blood meals were identified in October representing 5 species (Table 1). Chi-squared test showed significant temporal differences in the monthly proportion of blood meals from Green Heron (*p* < 0.0001), American Robin (*p* < 0.0001), and Common Yellowthroat (*p* < 0.0001) as the most frequent hosts for *Cs. melanura.*

### Frequency estimates of avian species and *Cs. melanura* blood feeding

Monthly frequencies of regional avian species in the study area are depicted in Fig. 2. Relatively higher frequencies of American Robin, Common Yellowthroat, Black-capped Chickadee, Red-eyed Vireo, Northern Cardinal, American Goldfinch, *Spinus tristis* L., and several other bird species were noticed throughout the year. The percentage of *Cs. melanura* that acquired blood meals from some of these bird species were as expected based on their abundance. However, no blood meals were identified from Blue Jay, *Cyanocitta cristata* L., Song Sparrow, *Melospiza melodia* Wilson, or Mourning Dove, *Zenaida macroura* L. despite the comparative abundance of these birds in the region, based on frequency estimates. Notably, Green Heron had a relatively lower frequency in the region, but comprised the most frequent source of blood meals for *Cs. melanura* in June and July.

### Eastern equine encephalitis virus infection in *Cs. melanura*

**Fig. 3:**
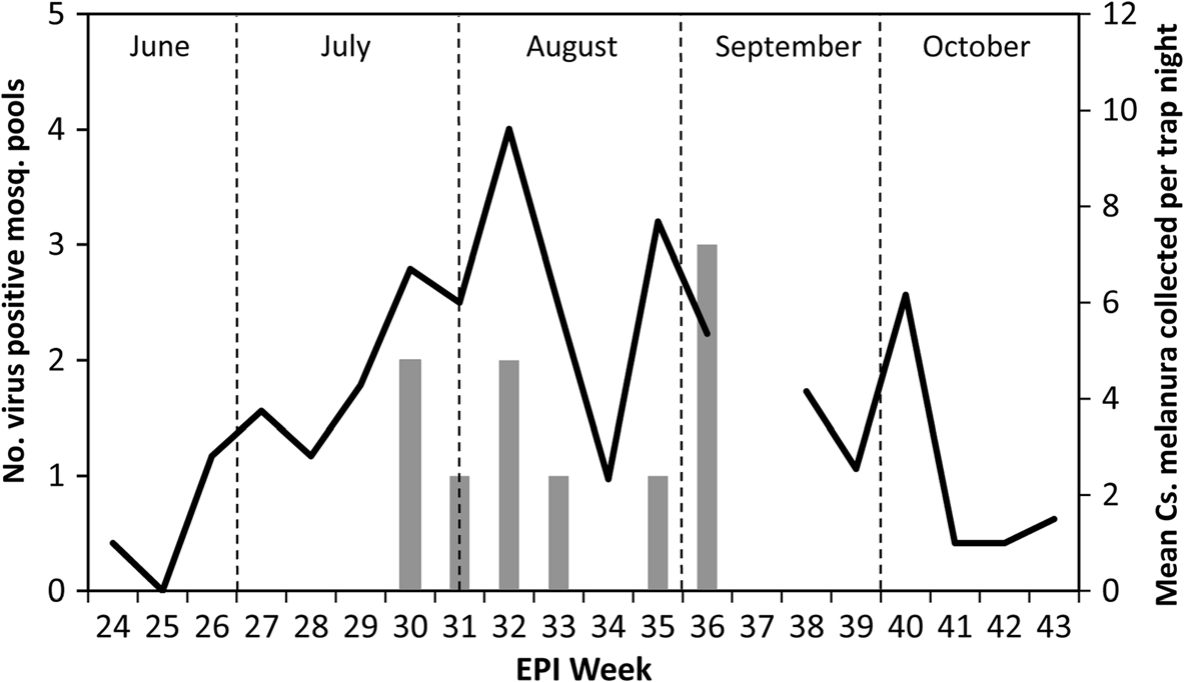
Weekly eastern equine encephalitis virus (EEEV) isolations and Maximum Likelihood Estimations (MLEs) compared to average *Cs. melanura* collected per trap night, 2012. The line graph represents the weekly average *Cs. melanura* captured per trap citywide. The bar graph represents the total number of EEEV-positive mosquito pools from each week with a corresponding MLE above the bar (calculated with both Vector Test and PCR positives of *Cs. melanura* pools)

Five virus isolates were recovered from head and thorax of individual blood-fed *Cs. melanura* in Vero cell culture, and all were subsequently identified as EEEV by real-time RT-PCR assays suggesting disseminated infection. These specimens had been collected on August 7, 2012, September 8, 2012 and September 18, 2012 from the towns of Whiting and Cornwall (Addison County), and Brandon (Rutland County). The host species for these mosquitoes were identified as American Robin, Common Yellowthroat, and Savannah Sparrow, *Passerculus sandwichensis* (Gmelin) (Table 4). EEEV was also detected from ten pools of non-blooded *Cs. melanura* from late July to early September (Fig. 3). The first EEEV positive pool was detected on July 24, 2012, and the last one on September 8, 2012. Mosquitoes were not collected during the second week of September (CDC week 37) due to an unexpected circumstance and unavailability of human resources. Virus-infected mosquitoes were collected from three trapping locations in close proximity to human and equine cases (Fig. 1).

**Table 4:** Identity of virus isolates from individual blood-fed *Cs. melanura* mosquitoes, date of collection, location, trap type, and blood meal sources, in Addison and Rutland Counties, Vermont, 2012

Date of collection	Location	Trap Type	Virus isolate	Blood meal source
8/7/2012	Whiting, Addison	Resting box	EEE virus	American Robin (*Turdus migratorius*)
9/8/2012	Cornwall, Addison	Resting box	EEE virus	Common Yellowthroat (*Geothlypis trichas*)
9/8/2012	Brandon, Rutland	Resting box	EEE virus	Common Yellowthroat (*Geothlypis trichas*)
9/8/2012	Whiting, Addison	Resting box	EEE virus	Common Yellowthroat (*Geothlypis trichas*)
9/18/2012	Whiting, Addison	Resting box	EEE virus	Savannah Sparrow (*Passerculus sandwichensis*)

### Phylogenetic analysis

**Fig. 4:**
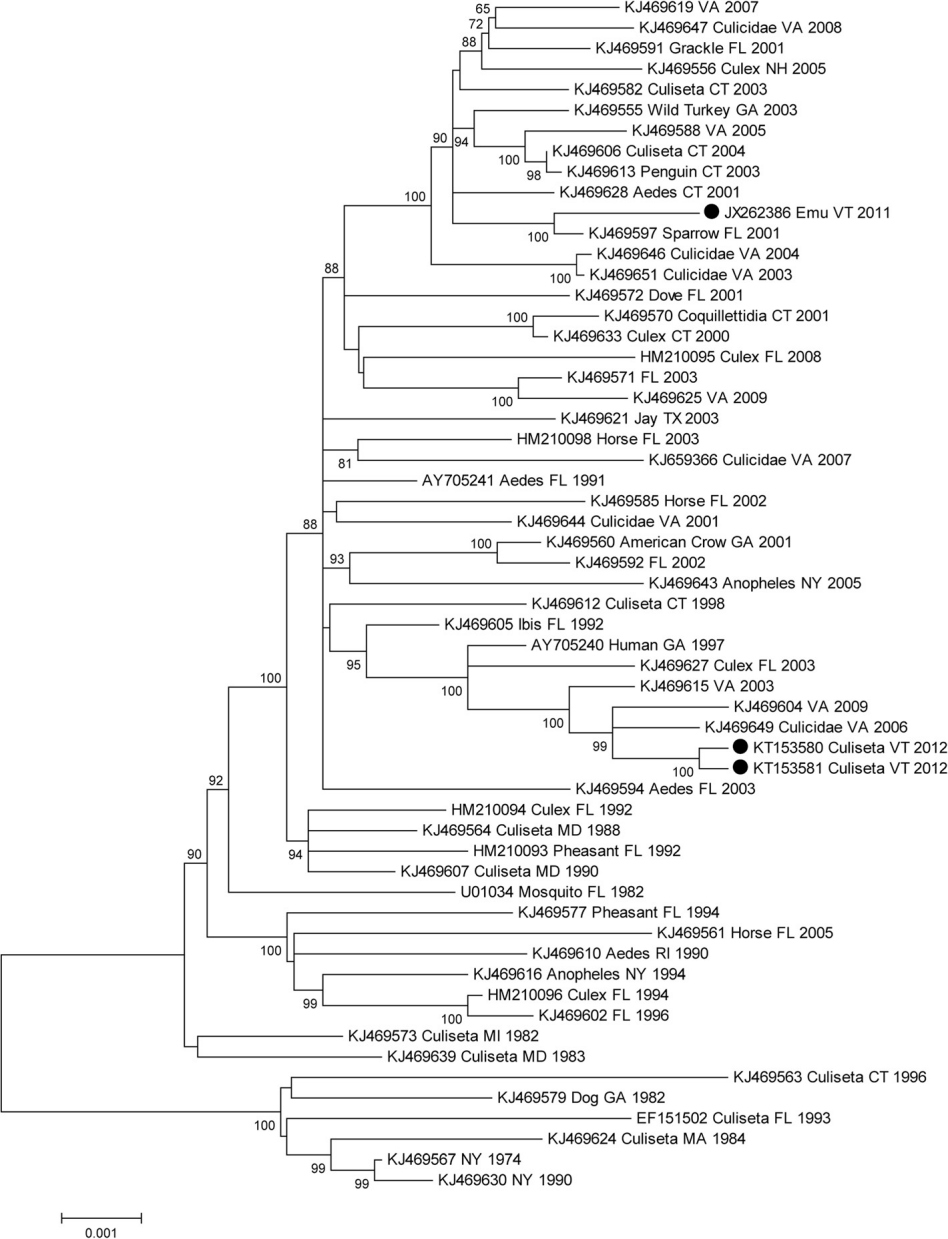
Maximum-likelihood tree based on EEEV sequences. Taxon names specify the GenBank number and state and year where they were collected. Numbers at nodes indicate bootstrap support >60 %. Branch lengths are proportional to the number of nucleotide subtitutions

To evaluate the phylogenetic relationships of EEEV isolates from Vermont mosquitoes, we sequenced two virus strains and compared these sequences with those available on GenBank. Maximum likelihood analysis revealed that Vermont strains from 2012 were most similar to each other yet distinct from an earlier isolate of EEEV in this state from 2011 (Fig. 4). The 2012 strains clustered with viruses isolated earlier in Virginia from 2003–2009 whereas the Vermont isolate from 2011 shared sequence identity with a Florida 2001 strain, as previously noted [18]. These findings suggest that EEEV was independently introduced into Vermont on at least two separate occasions.

## Discussion

Our analysis of the host associations of *Cs. melanura* provides further insight into the role of this species as the principal enzootic vector of EEEV in this northerly region of New England where the virus activity had been largely unrecognized. We found that *Cs. melanura* predominantly (94.3 %) feeds on passerine birds and focuses its feeding activity on several key species capable of supporting EEEV transmission, in particular, the Green Heron, American Robin, Common Yellowthroat, and Black-capped Chickadee. Our findings are consistent with previous studies from more southern geographic locales that have examined the host feeding patterns of *Cs. melanura* [10, 17, 21, 22, 41, 42]. However, a small percentage (5.7 %) of *Cs. melanura* was also identified with mammalian-derived blood meals including humans (1.2 %), suggesting occasional contribution of this species to epidemic transmission of EEEV in this region.

Green Heron is common and widespread from the northwestern U.S. across southeastern Canada and south to the West Indies, Panama, and northern South America, but may not be easily noticed (http://www.vermontbirds.org/vbba/pdf/VBBA1/Green%20Heron.pdf). They breed in coastal and inland wetlands and nest along swamps, marshes, lakes, ponds, impoundments, and other wetland habitats with trees and shrubs. In Vermont, the species is near the northern limit of its range. Therefore, its distribution is somewhat spotty, and it is absent or very irregular at higher elevations and in the northeastern corner of the state (http://www.vermontbirds.org/vbba/pdf/VBBA1/Green%20Heron.pdf). Green Heron arrives in Vermont around the third week in April and departs early in the fall, becoming scarce after early September (http://www.vermontbirds.org/vbba/pdf/VBBA1/Green%20Heron.pdf, http://www.allaboutbirds.org/guide/Green_Heron/id, https://en.wikipedia.org/wiki/Green_heron).

There is little information available on the competency of Green Heron for EEEV or its exposure status to the virus in the northeastern U.S. However, serological studies on Pelicaniformes and Ciconiiformes particularly Ardeidae family (Bitterns, Herons, and Allies) suggest these birds may be involved in amplification of EEEV in the southern U.S. In field studies conducted in Louisiana, Green Heron, Black-crowned Night Heron, *Nycticorax nycticorax* L.*,* Yellow-crowned Night Heron, *Nyctanassa violacea* L., and Great Blue Heron, *Ardea Herodias* L. were shown to have high prevalence of neutralizing antibody to EEEV, and the virus has been isolated from the blood of a nestling Yellow-crowned Night Heron [43–45]. Furthermore, the Snowy Egret, *Egretta thula* (Molina) has been shown to be highly susceptible to inoculation with EEEV, and neutralizing antibody was detected in this species from Florida [43, 45]. Recent blood meal analysis conducted in Florida has further revealed that these three herons are frequently fed upon by *Cs. melanura* [46]. Therefore, close association of Ardeidae birds with swamp habitats, where *Cs. melanura* and some other mosquito species breed, their ability to migrate over long distances and disperse following the breeding season, and high antibody prevalence suggest these birds could be involved in transport of EEEV from more southerly regions to the northeast and from further north to southern regions during fall migration [47].

American Robin served as the second most frequent host for *Cs. melanura* in the present study. Similarly, American Robin served as the most frequent source of blood meal for *Cs. melanura* in neighboring Massachusetts and Connecticut (22.9 %), and as the second most frequent host in New York (9.1 %) [10, 17, 21]. Resident and migratory populations of American Robin are common in North America, where they inhabit open and forested habitats in urban/suburban and rural settings, riparian forests, early successional forests, and closed canopy forests and woodlands [48]. Emergence of the first clutch of American Robin in early July in northern areas of the northeast temporally overlaps with the first generation of *Cs. melanura* [48–51] (http://www.epa.gov/housatonic/thesite/restofriver/reports/final_era/B%20-%20Focus%20Species%20Profiles/EcoRiskProfile_american_robin.pdf). American Robin is a moderately competent amplifying host, and EEEV has been isolated from local populations of this species in Massachusetts and New Jersey [2, 52, 53]. Therefore, results of our study in identifying American Robin as the second most frequent host in the present study, its abundance and other lines of evidence further suggest potential contribution of this avian species to maintenance and amplification of EEEV in the region.

As a New World warbler, Common Yellowthroat is active in open areas with thick, low and tangled vegetation, ranging from wetlands to grasslands to open pine forests and prairies. During migration, Common Yellowthroat uses diverse habitats including backyards and forests. The breeding range for Common Yellowthroat stretches across most of the United States, the Canadian provinces, and western Mexico (http://www.allaboutbirds.org/guide/Common_Yellowthroat/lifehistory). The Common Yellowthroat was identified as one of the most frequently fed upon hosts by *Cs. melanura* in upstate New York, and as a relatively frequent host in Connecticut and Massachusetts [10, 17, 21]. In serosurvey of wild birds for infection with EEEV, high antibody prevalence has been reported from Common Yellowthroat, or the virus has been isolated from this bird in New Jersey, New York, and Massachusetts [2, 53–56]. In a multi-year study of mosquito feeding patterns in a southeastern focus of EEEV in Alabama, Common Yellowthroat had a forage ratio estimate for *Cs. melanura* that was greater than one, suggesting that this bird species may be a preferred host [42]. In a serological study of wild birds for infection with *Alphaviruses* in upstate New York, Common Yellowthroat was antibody positive (*n* = 24, 19.0 %) for EEEV and the virus was isolated from five specimens (4.0 %) of this bird species [56].

As a small non-migratory songbird, Black-capped Chickadee lives in deciduous and mixed deciduous/coniferous woodlands, open woods, swamps, and dense canopies in North American (http://www.allaboutbirds.org/guide/Black-capped_Chickadee/lifehistory). Black-capped Chickadee is more common near edges of wooded areas, but can be found in the middle of large wooded tracts, and builds nests usually between 1.5 and 7 m high within the reach of questing mosquitoes. We identified Black-capped Chickadee as a frequent host of *Cs. melanura* in the present study in accordance with the findings of earlier investigations in upstate New York, Massachusetts, and Connecticut [10, 17, 21]. Black-capped Chickadee captured within EEEV foci in Massachusetts and New York have been reported to have very high antibody prevalence for EEEV, and the virus has been isolated from this bird [53, 56].

As a predominantly ornithophagic mosquito, the role of *Cs. melanura* in enzootic transmission of EEEV is well established. However, the potential contribution of this species to epidemic/epizootic transmission to humans/equines is unclear. Our study indicates that a small percentage of this mosquito species acquire blood meals from mammalian species including humans in support of other recent investigations [10, 17]. These findings in conjunction with its close association in time and space with virus foci, high virus titer in field-collected mosquitoes and physiological competence, local abundance, and frequent infection in nature, suggest that occasional human feeding by *Cs. melanura* could account for the comparatively few number of human cases that typically ensue periods of intense epizootic activity in this species.

In the present study, very small numbers of engorged *Ae. vexan*s and *Cq. perturbans* were captured and examined for the source of blood meals, therein precluding any possible assessment of their respective roles in transmission of EEEV in this region. However, EEEV has been repeatedly detected in *Cq. perturbans* in other regions of the northeast, highlighting its potential contribution to epidemic/epizootic transmission of EEEV where sufficient populations of this virus-competent species overlap temporally and spatially with *Cs. melanura* [8, 9, 24].

*Anopheles quadrimaculatus* and *An. punctipennis* contained mostly mammalian-derived blood meals with little inclination for feeding on birds. Other studies have identified a relatively greater percentage of avian-derived blood meals in these two Anopheline species, suggesting that they may occasionally acquire EEEV infection from viremic birds, and EEEV has been isolated from *An. quadrimaculatus* and *An. punctipennis* collected in northeastern U.S. [8, 22, 57–59]. Both are moderately competent vectors of EEEV, are abundant in wetland habitats where EEEV occurs, and seek hosts from mid-summer to early fall, when the virus is actively transmitted, thus suggesting that both *An. quadrimaculatus* and *An. punctipennis* may occasionally contribute to EEEV transmission in the northeastern U.S. [24].

Seasonal shifts in mosquito blood feeding from avian to mammalian hosts that may purportedly influence the role of various mosquito species in epidemic/epizootic transmission of arboviruses have been reported for other species [60–66]. Due to small percentage of *Cs. melanura* blood feeding from mammalian hosts, we did not observe a shift from avian to mammalian species; however, a late seasonal shift from Green Heron to American Robin, Common Yellowthroat, and Black-capped Chickadee was noted in August and September. The northern population of Green Heron moves to its breeding ranges during March and April, near the northernmost limit of this bird range, and breeding is well underway by the end of May. Migration to the winter quarters starts in September; by late October, these birds are absent from regions where they do not stay all year. Eastern breeders migrate via Florida, the Gulf Coast, and the Caribbean (http://www.vermontbirds.org/vbba/pdf/VBBA1/Green%20Heron.pdf, http://www.allaboutbirds.org/guide/Green_Heron/id, https://en.wikipedia.org/wiki/Green_heron). Although we have no direct evidence, these observations are consistent with the view that migrating Green Heron may contribute to early season introduction and “seeding” of EEEV into northern virus foci wherein other avian species subsequently serve as maintenance hosts and support viral amplification.

In this study, we recovered EEEV from the head and thorax portion of five *Cs. melanura* mosquitoes and identified their respective blood meal sources. These virus-infected mosquitoes had fed exclusively on passerine birds, specifically the Common Yellowthroat, American Robin, and Savannah Sparrow. The presence of virus in the head and thorax suggests that these were disseminated infections that were acquired from earlier blood meals than those identified in this study.

Phylogenetic analyses were also conducted to evaluate the geographic origin of EEEV strains from Vermont. Viral strains from 2012 were nearly identical to each other, but genetically distinct from a 2011 strain from Vermont. This suggests that EEEV was independently introduced into this geographic region on at least two separate occasions. The 2012 strain was most similar to EEEV isolated in Virginia from 2003–2009, whereas the 2011 strain grouped with a 2001 Florida strain, suggesting long-distance introduction of EEEV strains from southern source populations as previously discussed [11, 13].

## Conclusion

We show that *Cs. melanura* feeds primarily on passerine birds in Vermont and focuses its feeding activity on several species capable of supporting EEEV transmission, and occasionally acquires blood meals from mammalian hosts including humans. These behavioral characteristics, in conjunction with observations on its vector competence and high EEEV titers and infection rates in field-collected mosquitoes, qualify *Cs. melanura* not only as the principal vector of EEEV in enzootic cycle among wild bird species, but also enable this species to occasionally contribute to epidemic/epizootic transmission of EEEV in the region.

## References

[1] Morris CD, Monath TP (1988). Eastern equine encephalomyelitis. The Arboviruses: epidemiology and ecology.

[2] Crans WJ, Caccamise DF, McNelly JR (1994). Eastern equine encephalomyelitis virus in relation to the avian community of a coastal cedar swamp. J Med Entomol.

[3] Hayes RO, Beran GW (1981). Eastern and western encephalitis. Viral Zoonoses, Section B.

[4] Morris CD, Zimmerman RH (1981). Epizootiology of eastern equine encephalomyelitis virus in upstate New York, USA. III. Population dynamics and vector potential of adult *Culiseta morsitans* (Diptera: Culicidae). J Med Entomol.

[5] Scott TW, Weaver SC (1989). Eastern equine encephalomyelitis virus: epidemiology and evolution of mosquito transmission. Adv Virus Res.

[6] Howard JJ, Grayson MA, White DJ, Morris CD (1994). Eastern equine encephalitis in New York State. J FL Mosq Cont Assoc.

[7] Armstrong PM, Andreadis TG (2013). Eastern equine encephalitis virus-old enemy, new threat. N Engl J Med.

[8] Andreadis TG, Anderson JF, Tirrell-Peck SJ (1998). Multiple isolations of eastern equine encephalitis and highlands J viruses from mosquitoes (Diptera: Culicidae) during a 1996 epizootic in southeastern Connecticut. J Med Entomol.

[9] Centers for Disease Control and Prevention (CDC) (2006). Eastern equine encephalitis--New Hampshire and Massachusetts, August-September 2005. MMWR Morb Mortal Wkly Rep.

[10] Molaei G, Oliver J, Andreadis TG, Armstrong PM, Howard JJ (2006). Molecular identification of blood-meal sources in *Culiseta melanura* and *Culiseta morsitans* from an endemic focus of eastern equine encephalitis virus in New York. Am J Trop Med Hyg.

[11] Armstrong PM, Andreadis TG, Anderson JF, Stull JW, Mores CN (2008). Tracking eastern equine encephalitis virus perpetuation in the northeastern United States by phylogenetic analysis. Am J Trop Med Hyg.

[12] Rochlin I, Harding K, Ginsberg HS, Campbell SR (2008). Comparative analysis of distribution and abundance of West Nile and eastern equine encephalomyelitis virus vectors in Suffolk County, New York, using human population density and land use/cover data. J Med Entomol.

[13] Young DS, Kramer LD, Maffei JG, Dusek RJ, Backenson PB, Mores CN (2008). Molecular epidemiology of eastern equine encephalitis virus, New York. Emerg Infect Dis.

[14] Armstrong PM, Andreadis TG (2010). Eastern equine encephalitis virus in mosquitoes and their role as bridge vectors. Emerg Infect Dis.

[15] Gibney KB, Robinson S, Mutebi JP, Hoenig DE, Bernier BJ, Webber L (2011). Eastern equine encephalitis: an emerging arboviral disease threat, Maine, 2009. Vector Borne Zoonotic Dis.

[16] Lubelczyk C, Mutebi JP, Robinson S, Elias SP, Smith LB, Juris SA (2013). An epizootic of eastern equine encephalitis virus, Maine, USA in 2009: outbreak description and entomological studies. Am J Trop Med Hyg.

[17] Molaei G, Andreadis TG, Armstrong PM, Thomas MC, Deschamps T, Cuebas-Incle E (2013). Vector-host interactions and epizootiology of eastern equine encephalitis virus in Massachusetts. Vector Borne Zoonotic Dis.

[18] Saxton-Shaw KD, Ledermann JP, Kenney JL, Berl E, Graham AC, Russo JM (2015). The first outbreak of eastern equine encephalitis in Vermont: outbreak description and phylogenetic relationships of the virus isolate. PLoS One.

[19] Berl E, Eisen RJ, MacMillan K, Swope BN, Saxton-Shaw KD, Graham AC (2013). Serological evidence for eastern equine encephalitis virus activity in white-tailed deer, *Odocoileus virginianus*, in Vermont, 2010. Am J Trop Med Hyg.

[20] Mutebi JP, Swope BN, Saxton-Shaw KD, Graham AC, Turmel JP, Berl E (2012). Eastern equine encephalitis in moose (*Alces americanus*) in northeastern Vermont. J Wildl Dis.

[21] Molaei G, Andreadis TG (2006). Identification of avian- and mammalian-derived bloodmeals in *Aedes vexans* and *Culiseta melanura* (Diptera: Culicidae) and its implication for West Nile virus transmission in Connecticut. USA J Med Entomol.

[22] Apperson CS, Hassan HK, Harrison BA, Savage HM, Aspen SE, Faraji (Farajollahi) A (2004). Host-feeding patterns of established and potential mosquito vectors of West Nile virus in the eastern United States. Vector Borne Zoonotic Dis.

[23] Crans WJ, Schulze TL (1986). Evidence incriminating *Coquillettidia perturbans* (Diptera: Culicidae) as an epizootic vector of eastern equine encephalitis. I. Isolation of EEE virus from *Cq. perturbans* during an epizootic among horses in New Jersey. Bull Soc Vector Ecol.

[24] Vaidyanathan R, Edman JD, Cooper LA, Scott TW (1997). Vector competence of mosquitoes (Diptera: Culicidae) from Massachusetts for a sympatric isolate of eastern equine encephalomyelitis virus. J Med Entomol.

[25] Moncayo AC, Edman JD, Turell MJ (2000). Effect of eastern equine encephalomyelitis virus on the survival of *Aedes albopictus*, *Anopheles quadrimaculatus*, and *Coquillettidia perturbans* (Diptera: Culicidae). J Med Entomol.

[26] Cupp EW, Tennessen KJ, Oldland WK, Hassan HK, Hill GE, Katholi CR (2004). Mosquito and arbovirus activity during 1997–2002 in a wetland in northeastern Mississippi. J Med Entomol.

[27] Crans WJ, McNelly J, Schulze TL, Main A (1986). Isolation of eastern equine encephalitis virus from *Aedes sollicitans* during an epizootic in southern New Jersey. J Am Mosq Control Assoc.

[28] Moncayo AC, Edman JD (1999). Toward the incrimination of epidemic vectors of eastern equine encephalomyelitis virus in Massachusetts: abundance of mosquito populations at epidemic foci. J Am Mosq Control Assoc.

[29] Arrigo NC, Watts DM, Frolov I, Weaver SC (2008). Experimental infection of *Aedes sollicitans* and *Aedes taeniorhynchus* with two chimeric Sindbis/Eastern equine encephalitis virus vaccine candidates. Am J Trop Med Hyg.

[30] Sorenson E, Popp R, Lew-Smith M, Engstrom B, Lapin M, Ferguson M (2004). Hardwood swamps of vermont: distribution, ecology, classification, and some sites of ecological significance.

[31] Wood DM, Dang PT, Ellis RA. The mosquitoes of Canada (Diptera: Culicidae). Series. The insects and arachnids of Canada. Part 6. Biosystematics Res Inst Canada Dept Agric Publ. 1979;1686:390.

[32] Molaei G, Andreadis TG, Armstrong PM, Anderson JF, Vossbrinck CR (2006). Host feeding patterns of *Culex* mosquitoes and West Nile virus transmission, northeastern United States. Emerg Infect Dis.

[33] Molaei G, Andreadis TG, Armstrong PM, Diuk-Wasser M (2008). Host-feeding patterns of potential mosquito vectors in Connecticut, U.S.A.: molecular analysis of bloodmeals from 23 species of Aedes, Anopheles, Culex, Coquillettidia, Psorophora, and Uranotaenia. J Med Entomol.

[34] National Center for Biotechnology Information, The BLAST Sequence Analysis Tool. [http://blast.ncbi.nlm.nih.gov/Blast.cgi?PROGRAM=blastn&PAGE_TYPE=BlastSearch&LINK_LOC=blasthome].

[35] Armstrong PM, Prince N, Andreadis TG (2012). Development of a multi-target TaqMan assay to detect eastern equine encephalitis virus variants in mosquitoes. Vector Borne Zoonotic Dis.

[36] Zink SD, Jones SA, Maffei JG, Kramer LD (2013). Quadraplex qRT-PCR assay for the simultaneous detection of eastern equine encephalitis virus and West Nile virus. Diagn Microbiol Infect Dis.

[37] Kauffman EB, Jones SA, Dupuis AP, Ngo KA, Bernard KA, Kramer LD (2003). Virus detection protocols for West Nile virus in vertebrate and mosquito specimens. J Clin Microbiol.

[38] Larkin MA, Blackshields G, Brown NP, Chenna R, McGettigan PA, McWilliam H (2007). ClustalW and ClustalX version 2. Bioinformatics.

[39] Tamura K, Stecher G, Peterson D, Filipski A, Kumar S (2013). MEGA6: Molecular evolutionary genetics analysis version 6.0. Mol Biol Evol.

[40] Vermont Center for Ecostudies available online a: http://ebird.org/content/vt/

[41] Magnarelli LA (1977). Host feeding patterns of Connecticut mosquitoes (Diptera: Culicidae). Am J Trop Med Hyg.

[42] Estep LK, McClure CJ, Burkett-Cadena ND, Hassan HK, Hicks TL, Unnasch TR, Hill GE (2011). A multi-year study of mosquito feeding patterns on avian hosts in a southeastern focus of eastern equine encephalitis virus. Am J Trop Med Hyg.

[43] Kissling RE, Chamberlain RW, Nelson DB, Stamm DD (1955). Studies on the North American arthropod-Borne encephalitides. VIII. Equine encephalitis studies in Louisiana. Am J Hyg.

[44] Stamm DD (1958). Studies on the ecology of equine encephalomyelitis. Am J Public Health Nations Health.

[45] McLean RG, Crans WJ, Caccamise DF, McNelly J, Kirk LJ, Mitchell CJ, Calisher CH (1995). Experimental infection of wading birds with eastern equine encephalitis virus. J Wildl Dis.

[46] Bingham AM, Burkett-Cadena ND, Hassan HK, McClure CJ, Unnasch TR (2014). Field investigations of winter transmission of eastern equine encephalitis virus in Florida. Am J Trop Med Hyg.

[47] Spalding MG, McLean RG, Burgess JH, Kirk LJ (1994). Arboviruses in water birds (Ciconiiformes, Pelecaniformes) from Florida. J Wildl Dis.

[48] Martin K (1973). Breeding density and reproductive success of Robins in relation to habitat structure on logged areas of Vancouver Island, British Columbia.

[49] Hutto RL (1995). The composition of bird communities following stand-replacement fires in northern Rocky Mountain conifer forests. Conserv Biol.

[50] Sallabanks R. Effects of wildfire on breeding bird communities in coniferous forests of northwestern Oregon. Available online through Blue Mountains Natural Resources Institute at: http://www.fs.fed.us/pnw/mdr/past/bmnri/publications/abstract/sallabanks01.shtml.

[51] Poole A. The birds of North America; available online: Cornell Laboratory of Ornithology, Ithaca, NY. Available online at http://bna.birds.cornell.edu/BNA/2005/.

[52] Komar N, Dohm DJ, Turell MJ, Spielman A (1999). Eastern equine encephalitis virus in birds: relative competence of European starlings (*Sturnus vulgaris*). Am J Trop Med Hyg.

[53] Main AJ, Anderson KS, Maxfield HK, Rosenau B, Oliver C (1988). Duration of Alphavirus neutralizing antibody in naturally infected birds. Am J Trop Med Hyg.

[54] Emord DE, Morris CD (1984). Epizootiology of eastern equine en- cephalomyelitis in upstate, New York, USA. VI. Antibody prevalence in wild birds during an interepizootic period. J Med Entomol.

[55] Komar N, Spielman A (1994). Emergence of eastern encephalitis in Massachusetts. Ann N Y Acad Sci.

[56] Howard JJ, Oliver J, Grayson MA (2004). Antibody response of wild birds to natural infection with alphaviruses. J Med Entomol.

[57] Cupp EW, Stokes GM (1973). Identification of bloodmeals from mosquitoes collected in light traps and dog-baited traps. Mosq News.

[58] Irby WS, Apperson CS (1988). Hosts of mosquitoes in the coastal plain of North Carolina. J Med Entomol.

[59] Robertson LC, Prior S, Apperson CS, Irby WS (1993). Bionomics of *Anopheles quadrimaculatus* and *Culex erraticus* (Diptera: Culicidae) in the Falls Lake basin, North Carolina: seasonal changes in abundance and gonotrophic status, and host-feeding patterns. J Med Entomol.

[60] Tempelis CH, Reeves WC, Bellamy RE, Lofy MF (1965). A 3-year study of the feeding habits of *Culex tarsalis* in Kern County, California. Am J Trop Med Hyg.

[61] Edman JD (1971). Host-feeding patterns of Florida mosquitoes I. Aedes, Anopheles, Coquillettidia, Mansonia and Psorophora. J Med Entomol.

[62] Edman JD, Kale HW (1971). Host behavior: its influence on the feeding success of mosquitoes. Ann Ent Soc Am.

[63] Edman JD, Webber LA, Kale HW (1972). Host-feeding patterns of Florida mosquitoes. II. Culiseta. J Med Entomol.

[64] Edman JD, Webber LA, Schmid AA (1974). Effect of host defenses on the feeding pattern of *Culex nigripalpus* when offered a choice of blood sources. J Parasitol.

[65] Nelson RL, Tempelis CH, Reeves WC, Milby MM (1976). Relation of mosquito density to bird:mammal feeding ratios of *Culex tarsalis* in stable traps. Am J Trop Med Hyg.

[66] Thiemann TC, Wheeler SS, Barker CM, Reisen WK (2011). Mosquito host selection varies seasonally with host availability and mosquito density. PLoS Negl Trop Dis.

